# Assessment of common housekeeping genes as reference for gene expression studies using RT-qPCR in mouse choroid plexus

**DOI:** 10.1038/s41598-021-82800-5

**Published:** 2021-02-08

**Authors:** Kim Hoa Ho, Annarita Patrizi

**Affiliations:** 1 Schaller Research Group, German Cancer Research Center (DKFZ), DKFZ-ZMBH Alliance, Heidelberg, Germany; 2 Faculty of Biosciences, Heidelberg University, Heidelberg, Germany

**Keywords:** Developmental biology, Neuroscience

## Abstract

Choroid plexus (ChP), a vascularized secretory epithelium located in all brain ventricles, plays critical roles in development, homeostasis and brain repair. Reverse transcription quantitative real-time PCR (RT-qPCR) is a popular and useful technique for measuring gene expression changes and also widely used in ChP studies. However, the reliability of RT-qPCR data is strongly dependent on the choice of reference genes, which are supposed to be stable across all samples. In this study, we validated the expression of 12 well established housekeeping genes in ChP in 2 independent experimental paradigms by using popular stability testing algorithms: BestKeeper, DeltaCq, geNorm and NormFinder. *Rer1* and *Rpl13a* were identified as the most stable genes throughout mouse ChP development, while *Hprt1* and *Rpl27* were the most stable genes across conditions in a mouse sensory deprivation experiment. In addition, *Rpl13a*, *Rpl27* and *Tbp* were mutually among the top five most stable genes in both experiments. Normalisation of *Ttr* and *Otx2* expression levels using different housekeeping gene combinations demonstrated the profound effect of reference gene choice on target gene expression. Our study emphasized the importance of validating and selecting stable housekeeping genes under specific experimental conditions.

## Introduction

Choroid plexus (ChP) is a highly vascularized tissue located within the four brain ventricles. It is comprised of multiple cell types, including: epithelial, endothelial, mesenchymal and immune cells, with epithelial cells constituting the majority^1^. The epithelial layer encases connective stroma and a highly permeable fenestrated capillary network^2,3^, producing 70%–80% of the cerebrospinal fluid (CSF)^4,5^ and forming the blood-cerebrospinal fluid barrier (BCSFB)^4,5^. ChP is an understudied area in neuroscience but it is attracting more attention as its developmental function is gradually elucidated and its role in neuropathology is increasingly noticed^6^. Historically, ChP-CSF were only known to function as “cushion” (physical protection through buoyancy) and “sink” (removal of brain metabolites through CSF drainage) of the brain^4^. Recently, it has been demonstrated that CSF composition and ChP-derived factors, such as signalling and trophic molecules, play indispensable functions for brain development, brain homeostasis and adult neural stem cell niches. For example, it has been demonstrated that ChP has the intrinsic ability to sense external changes associated with CNS activity^7,8^ and it is also well known that ChP produces and releases Otx2, an essential factor implicated in both the onset and the closure of visual critical period^9^. In addition, ChP also controls the transportation of many blood-derived factors (nutrients, proteins, hormones, inorganic compounds, etc.) into the brain and it is emerging as the neuroimmune gateway regulating central nervous system (CNS) immune-surveillance^6^. Interestingly, changes in ChP-CSF structure and function have been linked to neurodegenerative diseases such as Alzheimer’s disease^10^, to neurodevelopmental disorders^11^ such as autism-spectrum disorder^12^ and schizophrenia^13^ and also to neuroimmune disease such as multiple sclerosis^14^. This increasing interest in ChP parallels the need for gene expression studies of ChP in different experimental contexts.

Despite the advances in high-throughput transcriptomic technologies like microarray and RNA sequencing^15^, reverse transcription quantitative real-time PCR (RT-qPCR) remains a popular method for measuring mRNA expression level, especially when the number of target genes is moderate. RT-qPCR is conceptually and technically simple, economical and fast yet still highly sensitive, accurate and reproducible^16^. Considered as the “gold standard” for gene expression analysis, RT-qPCR is utilised to validate microarray and RNA sequencing results^17^. Two major RT-qPCR quantification methods have been developed and are widely used: absolute and relative/comparative quantification. Absolute quantification allows the inference of transcript number from a standard/calibration curve, which is constructed as RT-qPCR fluorescence signals corresponding to serial dilutions of a known sample (cloned or synthetic cDNA). This method, therefore, relies on the externally-built curve and fails to consider inter- and intra-sample variabilities. Relative quantification addresses this limitation by measuring target gene expression relative to the expression of a reference gene within that sample. The ideal reference genes used in a RT-qPCR experiment are required to have constant expression levels regardless of biological differences and experimental conditions^18^. Housekeeping genes, constitutive genes required for the maintenance of basic cellular function, are therefore, often used for this purpose. However, such ideal reference genes have yet to be discovered^19^, which has led to a rise in literature evaluating the stability of reference genes specific for each species, tissue, cell type and condition of interest^20–24^.

To our knowledge, reference gene stability has not yet been assessed in ChP, potentially undermining RT-qPCR experiments on this tissue. Here, we selected and examined the stability of 12 well-established housekeeping genes: *Actb*, *Atp5f1* (also known as *Atp5pb*)*, B2m*, *Gapdh, Hprt1* (also known as *Hprt*)*, Pgk1, Rer1, Rpl13a, Rpl27, Sdha, Tbp, Ubc* in ChP of *Mus musculus* (house mouse). Using not only descriptive statistics but also a combination of 4 most popular stability assessment algorithms for reference genes: BestKeeper^25^, DeltaCq^26^, geNorm^18^ and NormFinder^27^, we tested the 12 reference genes in 2 experimental panels: Developmental and Light/Dark rearing panels and identified selective combinations of stable reference genes. Finally, we validated their effect as a normalisation factor to the expression of selective ChP markers, such as Transthyretin (*Ttr*)^28^ and Orthodenticle homeobox 2 (*Otx2*)^29^.

## Results

### Candidate reference genes, qPCR amplification experiment and descriptive statistics

The 12 candidate reference genes used in this study were selected based on their distinct cellular function and on their extensive use in neuroscience researches^18,22,23,30,31^. In particular, we selected genes belonging to different functional classes to reduce the possibility that their response to the same experimental condition is co-regulated. We examined genes involved in the cellular cytoskeleton (Actb), in transcription or translation (*Tbp, Rpl13a, Rpl27*), in cellular metabolism (*Gapdh, Sdha, Hprt1, Pgk1, Atp5f1*), and in protein degradation (*Ubc*) in addition to ubiquitous and common cellular components, such as the major histocompatibility complex class I component (*B2m*) and a structural membrane protein of the Golgi apparatus (*Rer1*). Detailed information for each primer pair is presented in Table 1. To ensure there was no undesired product during amplification, we first examined primer specificity in silico using NCBI PrimerBlast^32^ and later confirmed it by melting curve analysis. The results show one single sharp peak for each primer pair in wells containing cDNA and no signal in negative control wells, indicating target-specific amplification (Supplementary Figure S1).

**Table 1:** Summarized information of 12 candidate reference genes and 2 target genes. Tm: melting temperature (calculated by NCBI Primer-Blast with default settings for Primer Parameters).

Gene	Gene name (MGI)	Primer sequence (5′–3′)	Amplicon size (bp)	Tm	%GC	References
*Actb*	Actin, beta	TGACGTTGACATCCGTAAAG GAGGAGCAATGATCTTGATCT	143	56.83 56.17	45 42.86	^65,66^
*Atp5f1/Atp5pb*	ATP synthase peripheral stalk-membrane subunit b	GTCCAGGGGTATTACAGGCAA TCAGGAATCAGCCCAAGACG	112	59.44 59.75	52.38 55	^62,67^
*B2m*	Beta-2 microglobulin	TTCTGGTGCTTGTCTCACTGA CAGTATGTTCGGCTTCCCATTC	104	59.24 59.39	47.62 50	^68^
*Gapdh*	Glyceraldehyde-3-phosphate dehydrogenase	TGACCTCAACTACATGGTCTACA CTTCCCATTCTCGGCCTTG	85	58.59 58.21	43.48 57.89	^69–71^
*Hprt1/Hprt*	Hypoxanthine guanine phosphoribosyl transferase	CAAACTTTGCTTTCCCTGGT TCTGGCCTGTATCCAACACTTC	101	56.72 60.03	45 50	^72–74^
*Pgk1*	Phosphoglycerate kinase 1	TGGTGGGTGTGAATCTGCC ACTTTAGCGCCTCCCAAGATA	124	59.93 58.88	57.89 47.62	PrimerBank^75^ ID 70778975c3
*Rer1*	Retention in endoplasmic reticulum sorting receptor 1	GCCTTGGGAATTTACCACCT CTTCGAATGAAGGGACGAAA	137	57.77 55.79	50 45	^63,72,76^
*Rpl13a*	Ribosomal protein L13A	AGCCTACCAGAAAGTTTGCTTAC GCTTCTTCTTCCGATAGTGCATC	129	58.93 59.51	43.48 47.83	^77,78^
*Rpl27*	Ribosomal protein L27	AAGCCGTCATCGTGAAGAACA CTTGATCTTGGATCGCTTGGC	143	60.27 59.67	47.62 52.28	^72,76,79^
*Sdha*	Succinate dehydrogenase complex, subunit A, flavoprotein (Fp)	AGAAAGGCCAAATGCAGCTC GTGAGAACAAGAAGGCATCAGC	131	59.11 59.84	50 50	^41^
*Tbp*	TATA box binding protein	CCTTGTACCCTTCACCAATGAC ACAGCCAAGATTCACGGTAGA	119	58.92 59.1	50 47.62	^80,81^
*Ubc*	Ubiquitin C	GCCCAGTGTTACCACCAAGA CCCATCACACCCAAGAACA	104	59.89 57.26	55 52.63	^82,83^
*Ttr*	Transthyretin	CACCAAATCGTACTGGAAGACA GTCGTTGGCTGTGAAAACCAC	76	58.34 60.53	45.45 52.38	^84,85^
*Otx2*	Orthodenticle homeobox 2	TATCTAAAGCAACCGCCTTACG AAGTCCATACCCGAAGTGGTC	62	58.55 59.45	45.45 52.38	^86–88^

To better evaluate the stability of these housekeeping genes in various conditions, we established two experimental panels: (i) Developmental panel, consisting of ChP tissues from mice at postnatal day (P)0 (at birth), P15 (at eye opening), P30 (early puberty) and P60 (adulthood)^33^; (ii) Light/Dark rearing panel, consisting of ChP tissues from P60 mice reared in normal Light/Dark condition (Ctrl), completely in dark from birth (D), completely in dark then exposed to light for 1h (D-1hL), 4 h (D-4hL) and 24 h (D-24hL) (Supplementary Figure S2, Supplementary Table S1).

Due to the large amount of reactions per panel, each panel was divided into three 384-well plates with experimental setup following sample maximization approach, where each plate is prioritized to include all samples rather than all genes^34^. Inter-run calibration, therefore, is required to minimize variations between different runs (instrument-, reagent-, experimenter-related variations)^34^. Inter-run calibrators (IRC) were assigned to the amplification of *Gapdh* (Supplementary Figure S3).

We used quantification cycle (Cq) with the same meaning as cycle threshold (Ct ) or crossing point (Cp ) as recommended by RMDL consortium^35^. To acquire PCR efficiency (E) and coefficient of determination (R^2^) without standard curve, we used LinRegPCR^36^ which performs baseline correction for each sample individually and calculates E and R^2^ by fitting a regression line to a subset of data points in the log-linear phase. To combine the data of 3 plates from the same panel without inter-run variations, LinRegPCR output from 3 plates were then loaded into Factor-qPCR^37^, which determines the multiplicative factors and removes the systematic bias between different RT-qPCR runs. All data discussed below has been sequentially processed with both LinRegPCR and Factor-qPCR.

**Figure 1:**
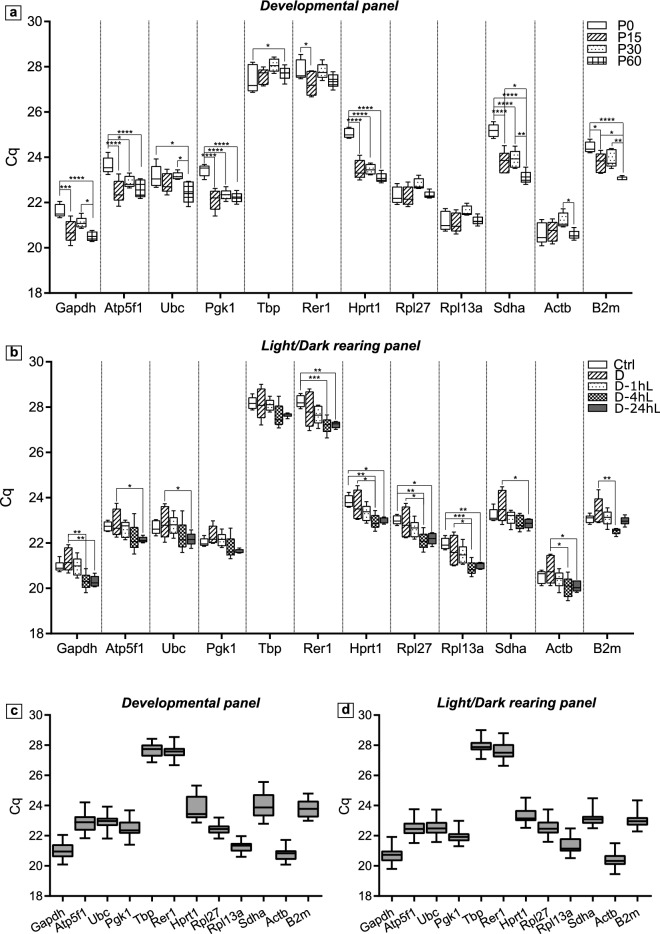
Descriptive statistics of 12 candidate reference genes in two sets of choroid plexus samples. Box-and-whisker plot showing Cq values of 12 housekeeping genes examined in the Developmental panel, displayed in different age groups (**a**) or displayed as all samples in the panel (**c**). Cq values of 12 housekeeping genes examined in the Light/Dark rearing panel, displayed in different rearing conditions (**b**) or displayed as all samples in the panel (**d**). Whisker indicates value range, the line inside the box was plotted at median value. (n = 5 mice per group (see Material and Method, Supplementary Table S1 for more details). Data is presented as Mean ± SD, adjusted p-values are indicated as *p ≤ 0.05, **p ≤ 0.01, ***p ≤ 0.001, ****p ≤ 0.0001. Cq: quantification cycle.

Descriptive statistics for the RT-qPCR amplification of both panels is presented in Table 2 and Fig. 1. The coefficient of determination (R^2^) shows how well the semi-log plot of Cq-log [cDNA concentration] fits to the linear regression model and indicates the presence of RT-qPCR inhibitors. We found that all primer pairs had R^2^ as 0.999 or 1.000, which is greater than 0.98 as recommended^38^ (Table 2). The amplification efficiency ranged within acceptable values, from 80.15 to 85.85 (Table 2). Mean Cq values and standard deviations (SD) were also calculated in Table 2. The range of Cq values (from 20.37 ± 0.49 to 27.93 ± 0.47) showed that all reference genes were expressed in ChP tissues (Table 2). We also confirmed the gene expression by in situ hybridization results from Allen Mouse Brain Atlas^39^. *Actb* was the most abundantly expressed gene in both experimental panels with the lowest mean Cq: 20.78 ± 0.44 in the Developmental and 20.37 ± 0.49 in the Light/Dark rearing panel, respectively. On the contrary, *Tbp* had the lowest expression level, which was reflected in its highest Cq of nearly 28. The genes that had the smallest and biggest variations were *Rpl27* (SD = 0.38) and *Sdha* (SD = 0.85) in the Developmental panel and *Pgk1* (SD = 0.41) and *Rer1* (SD = 0.58) in the Light/Dark rearing panel (Table 2).

**Table 2:** Descriptive statistics of Cq values and qPCR reactions. SD: standard deviation; R^2^: coefficient of determination; E: amplification efficiency; R^2^ and E were calculated using LinRegPCR software version 2020.0.

Gene	Developmental panel		Light/Dark rearing panel		R^2^	E (%)
	**Mean Cq**	SD	Mean Cq	SD		
*Actb*	20.78	0.44	20.37	0.49	0.999	80.25
*Atp5f1/ Atp5bp*	22.90	0.60	22.52	0.51	0.999	83.40
*B2m*	23.80	0.60	23.02	0.43	0.999	80.5
*Gapdh*	20.99	0.54	20.76	0.53	0.999	80.95
*Hprt1/ Hprt*	23.77	0.83	23.36	0.51	0.999	85.85
*Pgk1*	22.53	0.61	21.98	0.41	1.000	80.80
*Rer1*	27.57	0.47	27.64	0.58	1.000	80.15
*Rpl13a*	21.28	0.38	21.42	0.56	1.000	84.55
*Rpl27*	22.43	0.38	22.56	0.54	1.000	80.75
*Sdha*	23.99	0.85	23.15	0.47	0.999	80.35
*Tbp*	27.58	0.45	27.93	0.47	0.999	82.25
*Ubc*	22.91	0.49	22.57	0.56	0.999	80.70

Figure 1a, b visualised Cq values for each housekeeping gene at different postnatal ages and in specific rearing conditions. In the Developmental panel, Cq values of different age groups fluctuated differently depending on genes. In particular, Cq of *Gapdh, Atp5f1, Pgk1, Hprt1* and *Sdha* appear to be higher at P0 compared to later ages, whereas Cq values of the remaining housekeeping genes, did not show visible differences between P0 and later ages. Cq of *Atp5f1* and *Ubc* showed a large variation at P60, while the remaining genes appeared more consistent at this stage (Fig. 1a). In the Light/Dark rearing panel, Cq values of the 12 genes exhibited a common pattern across all the rearing conditions. All reference genes appear to be quite stable in the Ctrl and in the D-24hL conditions, whereas the D group showed a high variability across different genes. Generally, Cq values gradually decreased in the group order: Ctrl > D > D-1 h > D-4 h > D-24 h and this applied for all genes in the panel (Fig. 1b).

To better visualize how the expression of each gene varies within each experimental paradigm, we plotted Cq values of each gene as an entire set of samples per panel (Fig. 1c,d). These graphs showed that the variation of Cq values within each gene was maximum around 2 cycles per gene. Regardless of the experimental panels, Cq values for each gene appeared to fall into common ranges. In particular, *Tbp* and *Rer1* always have Cq between 26 – 29 whereas the other genes have the majority of Cq values between 20 – 25.

### Expression stability of candidate reference genes

To evaluate the expression stability of the 12 candidate genes, we applied the four most popular algorithms: BestKeeper^25^, DeltaCq^26^, geNorm^18^ and NormFinder^27^. We started by using RefFinder^40^, an online tool which incorporates all these algorithms and enables a fast, convenient analysis. However, some discrepancies were previously reported when comparing the stability results calculated by RefFinder and the original software of geNorm and NormFinder (BestKeeper and DeltaCq results were consistent)^20^. We, therefore, used the standalone geNorm (through qBase^34^ software) and NormFinder (R-based version) to verify RefFinder results, confirming minor differences in the stability ranking (Supplementary Table S2). All data discussed below were acquired using geNorm and NormFinder results through their original software whereas the BestKeeper and DeltaCq results were directly extracted from RefFinder.

**Figure 2:**
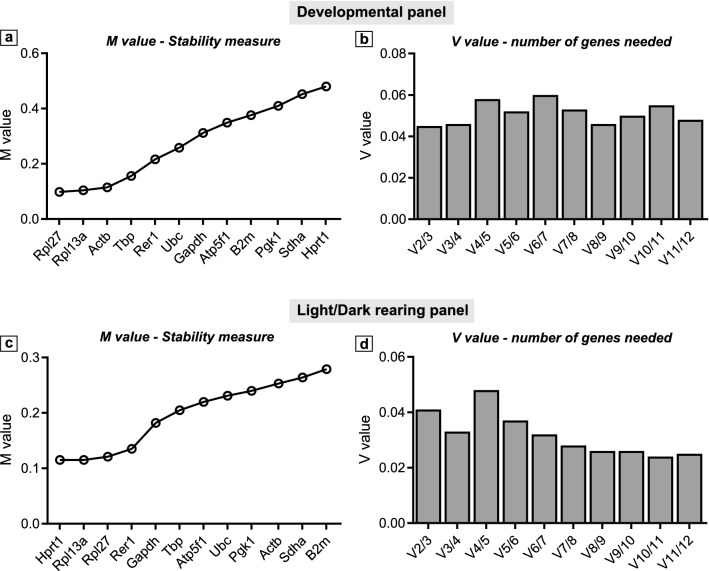
geNorm stability analysis. M value represents the gene expression stability in the Developmental (**a**) and in the Light/Dark rearing (**c**) panels. V value suggests the optimal number of reference genes per qPCR experiment in the Developmental (**b**) and in the Light/Dark rearing (**d**) panels.

geNorm algorithm calculates expression stability (M) and the ideal number of needed housekeeping genes (V) based on the concept that two ideal reference genes should have identical expression ratio in all samples, regardless of experiment conditions or tissue/cell line of origin^18^. A lower M value, calculated as average pairwise variation between one certain gene and other candidate reference genes, indicates a more stable expression. M lower than 0.5 is usually observed for stably expressed genes in homogeneous samples^34^. V value, displayed as V_n/n+1_, is the pairwise variation between two normalization factors. In brief, it calculates the necessity to include one more reference gene to the previous set of more stable reference genes. A V value greater than 0.15 suggests that the added gene has a significant effect and should be included for a more reliable normalisation factor^18^. In both experimental panels, M values were well below 0.5 for all reference genes demonstrating their stable expression in ChP. In the Developmental panel, *Rpl27* (M = 0.098) and *Rpl13a* (M = 0.104) were the two most stable genes, whereas *Sdha* (M = 0.452) and *Hprt1* (M = 0.48) were the least stable ones. In the Light/Dark rearing panel, *Hprt1* (M = 0.115) and *Rpl13a* (M = 0.115) were the two most stable genes; on the contrary *Sdha* (M = 0.264) and *B2m* (M = 0.279) were the least stable genes (Fig. 2a,c; Table 3). As all V values were below 0.15 and did not show significant difference within each panel, the use of the two most stable reference genes (*Rpl27, Rpl13a* in Developmental panel and *Hprt1, Rpl13a* in Light/Dark rearing panel) were considered sufficient (Fig. 2b,d).

**Table 3:** Stability values and rankings of ChP from the Development and the Light/Dark rearing panels calculated by four algorithms: geNorm, NormFinder, BestKeeper, DeltaCq and the overall ranking. Gene’s stability decreases from 1^st^ to 12^th^ rank.

Gene	Overall		geNorm		NormFinder		BestKeeper		DeltaCq	
	Geometric mean of rank (GMR)	Rank	Stability value (M)	Rank	Stability value (ρ)	Rank	Stability value (r)	Rank	Mean SD of mean $\Delta$Cq	Rank
**Developmental panel**										
*Rer1*	2.783	1	0.216	5	0.207	2	0.922	3	0.40	2
*Rpl13a*	2.783	= 1	0.104	2	0.309	6	0.965	1	0.45	5
*Gapdh*	2.817	2	0.312	7	0.161	1	0.662	9	0.39	1
*Rpl27*	3.600	3	0.098	1	0.314	7	0.900	4	0.45	6
*Tbp*	4.949	4	0.156	4	0.438	10	0.878	5	0.43	3
*B2m*	5.009	5	0.376	9	0.278	5	0.953	2	0.45	7
*Atp5f1*	6.126	6	0.349	8	0.261	4	0.651	11	0.44	4
*Actb*	6.620	7	0.115	3	0.351	8	0.656	10	0.47	8
*Ubc*	6.817	8	0.258	6	0.248	3	0.472	12	0.54	10
*Pgk1*	8.972	9	0.410	10	0.363	9	0.781	8	0.52	9
*Sdha*	9.453	10	0.452	11	0.469	11	0.878	6	0.60	11
*Hprt1*	10.487	11	0.480	12	0.473	12	0.816	7	0.62	12
**Light/Dark rearing panel**										
*Hprt1*	1.189	1	0.115	1	0.121	1	0.961	2	0.24	1
*Rpl27*	2.213	2	0.121	3	0.135	4	0.962	1	0.25	2
*Tbp*	2.711	3	0.205	6	0.132	3	0.949	4	0.26	3
*Rpl13a*	3.663	4	0.116	2	0.157	6	0.953	3	0.28	5
*Atp5f1*	4.527	5	0.220	7	0.123	2	0.940	5	0.29	6
*Gapdh*	5.318	6	0.182	5	0.151	5	0.962	= 1	0.31	8
*Ubc*	6.260	7	0.231	8	0.164	8	0.939	6	0.27	4
*Rer1*	6.817	8	0.135	4	0.189	10	0.939	= 6	0.32	9
*Pgk1*	7.454	9	0.240	9	0.163	7	0.917	7	0.30	7
*Actb*	9.212	10	0.253	10	0.166	9	0.899	8	0.33	10
*Sdha*	10.462	11	0.264	11	0.190	11	0.874	9	0.34	11
*B2m*	11.465	12	0.279	12	0.212	12	0.818	10	0.35	12

NormFinder applies the “model-based approach to estimation of expression variation”^27^ instead of pairwise comparison approach like the other methods. The algorithm estimates both intra- and inter-group variation, then combines them into the stability value ρ, which enables the addition of two sources of variation and confer a measure of systematic error. As a result, genes with smaller ρ exhibit higher stability^27^. In the Developmental panel, *Gapdh* was the most stable gene (ρ = 0.161) followed by *Rer1* (ρ = 0.207), whereas *Sdha* (ρ = 0.469) and *Hprt1* (ρ = 0.473) were the most inconsistent genes. For the Light/Dark rearing panel, *Hprt1* was the most stable gene (ρ = 0.121) followed by *Atp5f1* (ρ = 0.123). The least stable genes were *Sdha* (ρ = 0.190) and *B2m* (ρ = 0.212) (Table 3).

BestKeeper suggests that genes with SD greater than 1 are considered inconsistent^25^. Fortunately, all 12 genes studied have SD smaller than 1 and qualified for subsequent analysis. The algorithm computes Pearson’s correlation coefficient (r) between each gene and BestKeeper Index (geometric mean of Cp values), and genes with greater r values have more stable expression^25^. In the Developmental panel, the most stable genes were *Rpl13a* (r = 0.965) and *B2m* (r = 0.953) whereas the least stable genes were *Atp5f1* (r = 0.651) and *Ubc* (r = 0.472). In the Light/Dark rearing panel, the most stable genes were *Rpl27* (r = 0.962) and *Gapdh* (r = 0.962), there least stable genes were *Sdha* (r = 0.874 and *B2m* (r = 0.818) (Table 3).

The DeltaCq method, like geNorm, calculates pairwise comparisons of genes but with simpler mathematical calculations and without compromising accuracy. Specifically, it computes mean SD of differences in Cq ($\Delta$Cq) values of a given gene compared to all other genes in the studied list^26^. Genes with smaller mean SD have more stable expression. In the Developmental panel, the most stable genes by this method were *Gapdh* (Mean SD = 0.39) and *Rer1* (Mean SD = 0.40), the least stable genes were *Sdha* (Mean SD = 0.60) and *Hprt1* (Mean SD = 0.62). In the Light/Dark rearing panel, the range was smaller and the most stable genes were *Hprt1* (Mean SD = 0.24) and *Rpl27* (Mean SD = 0.25); at the bottom of the ranking were *Sdha* (Mean SD = 0.34) and *B2m* (Mean SD = 0.35) (Table 3).

**Figure 3:**
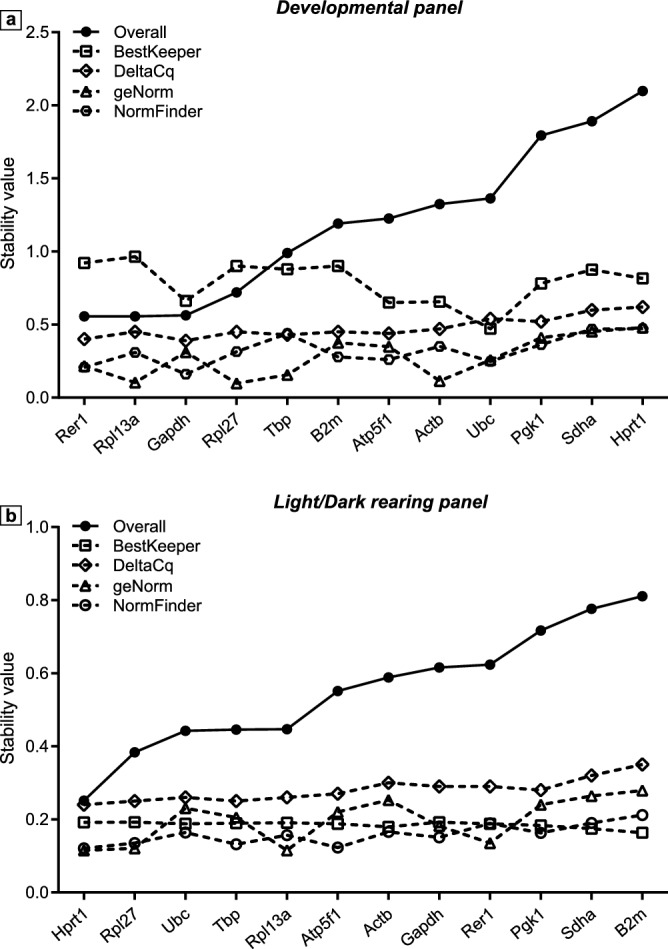
Conformity in rankings of reference genes by 4 methods. Stability ranking of 12 reference genes in the Developmental (**a**) and in the Light/Dark rearing (**b**) panels, calculated per assessment method (geNorm, triangle; NormFinder, hexagon; BestKeeper, square; DeltaCq, diamond) and as geometric mean of the 4 rankings (Overall, full black circle) (see Table 3 for more details).

To visually illustrate how consistent the results of the described four algorithms are, we plotted the graph of genes and stability values in Fig. 3 with lower stability values indicating more stable expression. In general, the variations in gene stability between methods appeared to be smaller in the Light/Dark rearing panel than in the Developmental panel. When comparing stability rankings calculated by the 4 methods, the results were more consistent at both ends of the chart (Light/Dark rearing panel) or within the lower values (Developmental panel). The middle of both graphs was slightly fluctuating, indicating that the algorithms are less consistent to each other when the difference in the gene stability is less significant. This inconsistency in stability ranking of the 4 methods (Table 3, Fig. 3) is the result of differences in either mathematics or in the concepts of gene stability on which the algorithms were established. Similar inconsistencies have also been reported in other studies^20,24,30,41,42^.

To draw a conclusion on stability testing results, overall ranking was determined by calculating geometric mean of rankings (GMR) from all four methods (Table 3, Fig. 3), with smaller GMR indicating more stable expression. We identified *Rer1* (GMR = 2.783) and *Rpl13a* (GMR = 2.783) as the two most stable genes in the Developmental panel, whereas *Sdha* (GMR = 9.453) and *Hprt1* (GMR = 10.847) were the two least stable genes. In the Light/Dark rearing panel, the two most stable genes were *Hprt1* (GMR = 1.189) and *Rpl27* (GMR = 2.213), whereas the two least stable ones were *Sdha* (GMR = 10.462) and *B2m* (GMR = 11.465) (Table 3).

Altogether these data suggest that the expression level of frequently used housekeeping genes could vary depending on different experimental conditions, supporting the notion that a preliminary study to find optimal reference genes should be performed whenever possible^20,21,30,43^.

**Figure 4:**
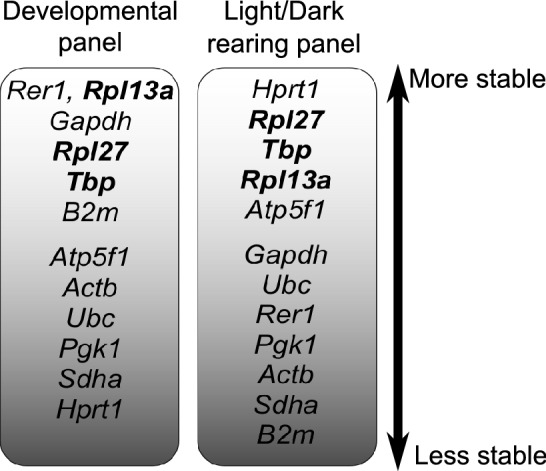
Summary of gene stability ranking. The 2 columns illustrate gene stability order of the 2 experimental panels with decreasing stability from top to bottom. Three genes (bold) were among the top 5 most stable for both panels.

The stability ranking of genes in both panels is summarised in Fig. 4, which was divided into the five top ranked genes and the rest. Within the five top ranks, we identified three housekeeping gene candidates, *Tbp, Rpl13a* and *Rpl27*, which were considered relatively stable across the two studied conditions and would also be used for the following validation experiments.

### The effect of different reference genes on relative expression of target genes

To determine the impact of reference gene choice on gene expression results, we used different combinations of housekeeping genes to calculate the expression of *Ttr* in the Developmental panel, and *Otx2* in the Light/Dark rearing panel. *Ttr*, encoding transporter protein transthyretin (formerly called prealbumin), is a ChP marker^28^ and its expression is expected to change among groups in the Developmental panel. Orthodenticle homeobox 2 (*Otx2*), is a homebox gene encoding for a transcription factor synthesized and secreted by ChP^29^, which has proven roles in regulating critical period in the visual system^44–47^. We, therefore, selected *Otx2* as a good target gene for the Light/Dark rearing panel.

**Figure 5:**
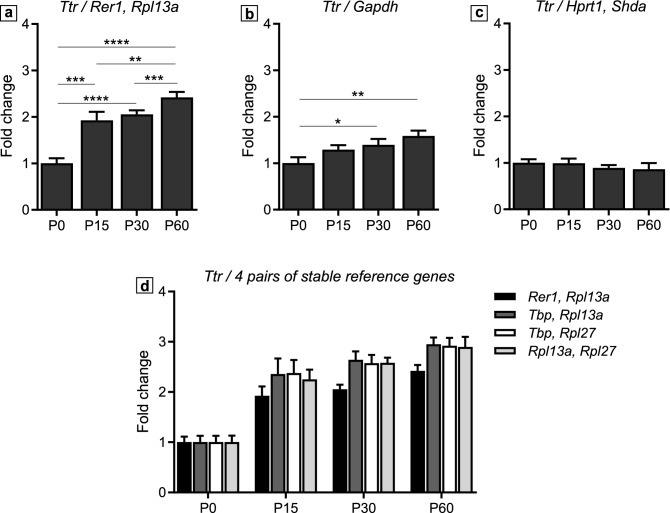
Relative expression of *Ttr* normalised to different reference genes. *Ttr* relative expression compared to the most stable reference genes in the Developmental panel: *Rer1, Rpl13a* (**a**); to *Gapdh* (**b**) and to the least stable genes in the Developmental panel: *Sdha, Hprt1* (**c**). *Ttr* relative expression normalized to *Rer1, Rpl13a* versus different combination of the 3 common stable genes *Tbp, Rpl13a, Rpl27* (**d**). Data is presented as Mean ± SD, adjusted p-values are indicated as *p ≤ 0.05, **p ≤ 0.01, ***p ≤ 0.001, ****p ≤ 0.0001.

For averaging the reference genes, we used geometric mean rather than arithmetic mean as it is suggested to offer better control over possible outliers and abundance differences between different genes^18^. We normalised *Ttr* expression to the two most stable genes in Developmental panel, *Rer1*, *Rpl13a* (Fig. 5a), to the most common reference gene, *Gapdh* (used in many ChP studies^48–50^) (Fig. 5b), to the two least stable genes in the same panel, *Sdha*, *Hprt1* (Fig. 5c), and to different combinations of any two among three stable genes, *Rpl13a*, *Rpl27*, *Tbp* (Fig. 5d). Normalization with *Rer1* and *Rpl13a* (the most stable) nearly doubled the *Ttr* relative expression level between P0 and P15, followed by a steadier rise until P60 (Fig. 5a). Although it is still possible to see the increase in *Ttr* expression throughout development with the commonly used reference *Gapdh*, the difference was less obvious and less statistically powerful. In addition, the statistical difference between P0 and P15 was lost (Fig. 5b). When the least stable pair *Sdha* and *Hprt1* was used, *Ttr* relative expression level was flattened and there was no difference between the four developmental stages (Fig. 5c). Finally, Fig. 5d shows the comparison between the best developmental reference genes [*Rer1, Rpl13a*] and 3 pairs of good reference gene *pan*-panels [*Rpl13a, Rpl27*]*,* [*Rpl13a, Tbp*] and [*Rpl27, Tbp*]. Although with moderately higher fold change expression of *Ttr,* the later 3 pairs gave highly similar results compared to using [*Rer1, Rpl13a*] (Fig. 5d), which demonstrates their suitability for being used as suboptimal reference genes in the Developmental panel. Finally, we tested whether using *Gapdh* in combination with another more stable gene, namely *Rer1, Rpl13a* or *Tbp,* would improve the normalization of *Ttr* mRNA expression. Despite relatively lower fold change values, the results showed a good “rescue effect” with expression patterns similar to those seen by normalising to the most stable genes [*Rer1, Rpl13a*] (Supplementary Fig. S4a).

**Figure 6:**
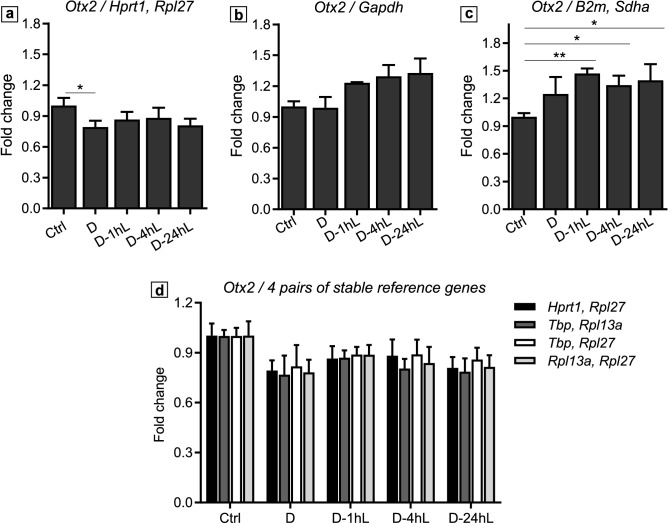
Relative expression of *Otx2* normalised to different reference genes. *Otx2* relative expression compared to the most stable reference genes in the Light/Dark rearing panel: *Hprt1, Rpl27* (**a**); to *Gapdh* (**b**) and to the least stable genes in the Light/Dark rearing panel: *B2m, Sdha* (**c**). *Otx2* relative expression normalized to *Hprt1, Rpl27* versus different combination of the 3 common stable genes *Tbp, Rpl13a, Rpl27* (**d**). Data is presented as Mean ± SD, adjusted p-values are indicated as *p ≤ 0.05.

Similarly, in the Light/Dark rearing panel when we normalized *Otx2* expression to *Hprt1* and *Rpl27*, the two most stable genes, we noticed a reduction of *Otx2* expression in the D group compared to the Ctrl group. However, *Otx2* level was steady across all dark rearing conditions, D, D-1hL, D-4hL and D-24hL (Fig. 6a). Normalisation to *Gapdh* gave heavily distorted results with *Otx2* expression levels unchanged between the Ctrl and the D samples, and increased expression in D-1hL, D-4hL and D-24hL samples (Fig. 6b). Strikingly, when *Sdha* and *B2m* (the least stable genes) were used *Otx2* expression was significantly increased in all dark rearing groups (D, D-1hL, D-4hL and D-24hL) compared to the Ctrl group. *Otx2* mRNA level was highest at D-1hL (Fig. 6c). Finally, Fig. 6d displays the comparison between the best genes in the Light/Dark rearing panel [*Hprt1, Rpl27*] and 3 pairs of good reference gene *pan*-panels [*Rpl13a, Rpl27*]*,* [*Rpl13a, Tbp*] and [*Rpl27, Tbp*]. Although there were some differences in the results, the overall trend of *Otx2* mRNA expression level was maintained across different experimental groups, which shows their competency as suboptimal reference genes for this panel. Again, we tested whether using *Gapdh* in combination with another more stable gene, namely *Hprt1, Rpl27* or *Tbp,* would improve the normalization of *Otx2* mRNA expression. Unlike the case of *Ttr,* the addition of 1 more stable gene to the reference pair did not improve *Otx2* measurement to the pattern obtained using the most stable genes [*Hprt1, Rpl27*] (Supplementary Fig. S4b), which could be due to the difference in the stability rankings of *Gapdh* between the 2 panels. In fact, *Gapdh* ranked 2^nd^ in the Developmental panel and 6^th^ in the Light/Dark rearing panel, which suggests that its expression might be affected by different light/dark rearing regimes (Fig. 4).

Altogether, these data emphasized the significant impact of reference gene choice on the expression levels of target genes. Our data also suggest that *Rpl13a*, *Rpl27* and *Tbp* are relatively stable housekeeping genes in ChP across two experimental paradigms and that any combination among them has the potential to be a good choice of reference genes for ChP.

## Discussion

ChP is emerging as an underestimated but important brain tissue, playing crucial roles in development, homeostasis and protection of the CNS. Therefore, the growing body of ChP research would benefit from reproducible and accurate protocols for measuring ChP gene expression. For RT-qPCR, this requires reference genes whose expression is stable across developmental stages and/or treatment conditions. In this study we investigated the expression stability of 12 common housekeeping genes (*Actb, Atp5f1, B2m, Gapdh, Hprt1, Pgk1, Rer1, Rpl13a, Rpl27, Sdha, Tbp* and *Ubc*) in ChP across developmental stages (Developmental panel) or brain activity (Light/Dark rearing panel). We analysed expression data of housekeeping genes using both descriptive statistics (Table 2, Fig. 1) and more sophisticated analysis by implementing specific algorithms built on different perceptions of housekeeping gene stability (Table 3, Figs. 2, 3). Our data indicate that different experimental paradigms require selective reference genes, confirming the importance of identifying suitable reference genes in relative quantitative RT-qPCR.

An initial evaluation of the housekeeping gene stability using descriptive statistics, measuring mean Cq and SD already highlighted variations between groups in the same panel and across panels (Fig. 1). For instance, Cq values for *Tbp*, *Rer1, Rpl13a* and *Rpl27* were relatively stable across developmental stages, while *Atp5f1*, *Hprt1*, *Pgk1* and *Sdha* showed significantly higher Cq for P0 versus older ages. This method is usually a tempting way of evaluating gene stability due to its simplicity but it is valid only to a certain extent. In fact, its apparent simplicity belies the biological and technical variations. In order to find a more robust measure of gene stability, we employed 4 widely used application packages: geNorm^18^, BestKeeper^25^, DeltaCq^26^, and NormFinder^27^. Differences in the methodology used by each algorithm may cause a few inconsistencies and make the result “relative” (Table 3, Fig. 3). For example, in the Light/Dark rearing panel, all algorithms agreed that *Sdha* and *B2m* were the least stable genes, 3 of the 4 algorithms ranked *Hprt1* as the most stable gene but *Rpl27* received 4 different rankings from the 4 methods (although all within the top 4) (Table 3). Therefore, in order to incorporate results from all 4 methods, we took geometric means of the 4 rankings to generate overall final stability values, which conclude that *Rer1, Rpl13a* were the 2 most stable genes in the Developmental panel while *Hprt1 and Rpl27* were the 2 most stable genes in the Light/Dark rearing panel.

Another important issue to take into consideration is the number of reference genes that is sufficient to obtain reliable results without wasting materials. The use of one single reference gene is generally not advisable as Vandesompele et al. (2002) showed that normalisation using only one reference gene would lead to erroneous results up to 3.0 fold in 25% of cases and 6.4 fold in 10% of cases^18^. geNorm’s V values demonstrated that the optimal number of reference genes for ChP in each experimental panel is 2 when selected from the most stable genes (Fig. 2b, d).

We also noticed that a subset of genes, *Rpl13a, Rpl27* and *Tbp* were among the top 5 most stable genes in both panels (Fig. 4). Interestingly, the use of a combination of these genes across the two panels was enough to maintain the expression pattern as observed when using the most stable genes. In fact, the normalized expression level of *Ttr* and *Otx2* was comparable when using the most stable pairs for each panel or a combination of 2 among the 3 commonly stable genes, *Rpl13a, Rpl27, Tbp*, demonstrating that these are suboptimal but acceptable reference genes in both panels (Figs. 5d, 6d). On the contrary, when comparing the expression level normalized to the most stable, the least stable, and *Gapdh,* we always visualized a stark contrast, showing how significant the choice of reference genes could alter target gene expression readings (Figs. 5a–c, 6a–c).

Standing out as commonly stable genes in both panels, *Tbp, Rpl13a* and *Rpl27* are all involved in transcription/translation machinery. While *Tbp* encodes TATA-box binding protein – an important part of the eukaryotic transcription initiation complex^51^, *Rpl13a* and *Rpl27* encode ribosomal protein L13a and L27, which are components of the 60S ribosome^52^. Although it is not always the case, *Tbp* and ribosomal proteins often appeared as stable candidates in many other reference gene analyses^18,42,53^. However, despite their high stability rankings, we do not recommend a reference set only containing *Rpl13a* and *Rpl27* as they belong to the same family and may be subject to co-regulation. On the other side, genes involved in cellular metabolism, such as *Atp5f1* (encoding subunit B in peripheral stalk of mitochondrial ATP synthase)^54^, *Sdha* (encoding an enzyme of tricarboxylic acid cycle)^55^, *Pgk1* and *Gapdh* (encoding enzymes of the glycolytic pathway)^56,57^ appear to be quite unstable in ChP across the conditions examined, suggesting that they may be modulated by the used experimental paradigms. In general, metabolic activities have been shown to change in response to external manipulation, such as photic manipulations^58^ and developmental stages^59^, which may explain why *Atp5f1, Sdha, Pgk1* and *Gapdh* expression is relatively unstable in this study and tend to rank at the bottom. Another common family of reference gene includes structural genes, such as *Actb* (encoding actin protein of cytoskeletal structure) and tubulin (encoding microtubules). It is well-known that tubulin genes are essential for the development and function of neurons^60^, however it has been reported that tubulin mRNA can be unstable under physiological changes^61^; therefore, we decided to not include it in our study. On the other hand, we did include *Actb*, one of the most popular gene in the literature. However, in our experimental condition it only ranked 7^th^ in the Developmental panel and 10^th^ in the Light/Dark rearing panel, which is in agreement with many other studies showing that structural cellular components, such as *Actb* are not stable housekeeping genes and therefore not good candidate reference genes^30,41,62^.

Finally, *Gapdh*, the most popular housekeeping gene in the literature, was not an ideal candidate in our study either, where it ranked 2nd in the Developmental panel and 6th in the Light/Dark rearing panel. *Gapdh* instability could be attributed to the fact that it is a metabolic gene and sensitive to our study’s experimental conditions as mentioned above. Despite its abundance in ChP homogenate, the mediocre quality of *Gapdh*, as a reference gene, was reflected in the RT-qPCR experiment with *Ttr* (Fig. 5a, b), in which the increase in *Ttr* expression across development was observed but with a reduced statistical significance, especially at early developmental stages (P0-P15). In the Light/Dark rearing panel, where *Gapdh* was found in a lower stability ranking (Fig. 4), the normalization to *Gapdh* even gave a false sense of *Otx2* expression compared to most stable genes in the same panel (Fig. 6a, b). In agreement with several previous studies^23,30,41,42^, we noticed that *Gapdh* may not be a reliable reference gene for the analysis of ChP. Here, we suggest that its use should be considered in specific experimental context and be combined with or even replaced by other housekeeping genes when appropriate.

In summary, we recommend the use of a minimum of 2 reference genes for ChP RT-qPCR. In particular, *Rer1* and *Rpl13a* appear to be stable in the ChP across postnatal ages, whereas *Hprt1* and *Rpl27* are mainly stable when neuronal activity is manipulated. We also showed that *Rpl13a*, *Rpl27* and *Tbp* were relatively stable genes across both experimental conditions in this study. Our results demonstrated that the expression of housekeeping genes in ChP could change depending on experimental settings and that the choice of reference genes can have a great impact on the measured expression levels of target genes. Therefore, it is worthwhile to investigate the stability of candidate reference genes in the context of the specific experiment (i.e. species, cell types, treatment, etc.) whenever possible.

## Materials and methods

### Selection of candidate reference genes and primers

14 genes were examined, including 12 housekeeping genes and 2 target genes. All primer pairs were adapted from the literature (Table 1) with the following criteria: primer length 18–24 bp, amplicon size 50–150 bp, melting temperature (Tm) 56–60 °C, GC content 40–60%, no secondary structures formed at annealing temperature (56 °C). Primer specificity was checked in silico by NCBI-PrimerBlast^32^. Primers were synthesised by Sigma Aldrich.

### Animal samples collection

All animal experiments were approved by the local governing regional council (*Regierungspräsidium Karlsruhe*, Germany). All methods were carried out following the German Animal Welfare Act regulations. Animal studies are reported in compliance with the ARRIVE guidelines.

Inbred C57Bl6/J mice were purchased from Janvier labs. Light/Dark rearing mice were socially housed in groups of two to five animals. All mice were housed in static cages. No environmental enrichment toys were added to the cages, only extra Kimwipes tissue. Light-reared mice were maintained on a 12 hour light/dark cycle, whereas dark-reared litters were kept in complete darkness from birth until adulthood (P60). A subset of them was re-exposed to light for different times (Supplementary Fig. S1). Mice were housed in standard housing conditions and received ad libitum food and water.

Newborn pups (P0) were decapitated while animals at P15, P30 and P60 were sacrificed by cervical dislocation. Both male and female animals were included. Freshly harvested brains were kept in Harvesting media^63^ (DMEM-F12 supplemented with 10% FBS, 2 mM L-Glutamine, 50 μg/ml Gentamycin (all reagents are from Gibco)) on ice, then ChP tissue was isolated from lateral and fourth ventricles, pooled together and immediately frozen in liquid nitrogen and stored at − 80 °C. ChP isolation was performed on ice and supported by stereomicroscope Stemi DV4 (Zeiss).

### Total RNA isolation and cDNA synthesis

Total RNA was extracted from ChP tissue using RNeasy Mini Kit (Qiagen 74,104) following manufacturer’s protocol. Tissue was disrupted and homogenised using Lysis buffer RTL and QIAshredder spin columns (Qiagen 79,654). Genomic DNA was removed using RNase-free DNase I (Qiagen 79,254) for 15 min at room temperature. RNA was eluted from the column with 30 µl RNase free water and stored at − 80 °C. RNA quality and concentration were measured by UV spectrophotometry on NanoDrop One (Thermo Scientific). RNA yield for each biological sample ranged from 800 to 3300 ng. The desired absorbance ratios A_260_/A_280_ and A_260_/A_230_ were 1.8–2.2. RNA samples with absorbance ratios below 1.8 were precipitated using 100% ethanol, ammonium acetate and glycogen (Thermo Scientific R0551) following manufacturer’s protocol. For cDNA synthesis, 270–990 ng RNA each sample was used with High-Capacity RNA-to-cDNA kit (Thermo Scientific 4,387,406). cDNA samples were stored at − 20 °C.

### Reverse transcription quantitative real-time PCR (RT-qPCR) experiment

For expression stability of housekeeping genes experiment, each panel was divided into two 384-well plates with experimental setup following the sample maximization approach^34^. In the Developmental panel, there were 4 age groups, 5 biological replicates per group (sample number n = 20). In the Light/Dark rearing panel, there were 5 groups, 5 biological replicates each group (sample number n = 25) (Supplementary Table S1 ). Within each panel, the amplification of *Gapdh* in 4–5 samples were assigned as inter-run calibrators (IRC)^34^ (see Supplementary Fig. S3 for the plate layout). For the RT-qPCR experiment of target genes’ expression—*Ttr* and *Otx2—*with different normalisation factors, all reactions for each gene were carried out on one single 384-well plate, 3 biological replicates each group. The number of technical replicates was 3 for all reactions. No template controls (NTC) were included in all experiments.

RT-q PCR was performed using PowerUp SYBR Green Master Mix (Thermo Scientific A25742) following manufacturer’s protocol with 50 ng cDNA, 20 µM each primer in a 10 µl reaction volume. Thermal protocol consisted of UDG activation at 50 °C in 2 min, Dual-Lock DNA polymerase activation at 95 °C in 2 min, 40 cycles of Denaturation at 95 °C in 15 s, Annealing at 56 °C in 15 s and Extension at 72 °C in 1 min. Melting curve thermal protocol was run right after finishing amplification: 95 °C in 15 s (ramp rate 1.6 °C/s), 60 °C in 1 min (ramp rate 1.6 °C/s), 95 °C in 15 s (ramp rate 0.15 °C/s). All RT-qPCR experiments were performed on LightCycler 480 (Roche).

### Raw data processing

Non-baseline-corrected RT-qPCR raw data was extracted from the machine to provide input for LinRegPCR^36^ (version 2020.0). The software performed baseline correction for each sample individually, calculated amplification efficiency (E), quantification cycle (Cq) and coefficient of determination (R^2^) by fitting a linear regression model to log-linear phase. Technical replicates were next examined with the allowed maximum variation of Cq as 0.5 (default threshold in qBase^34^), unqualified replicate was eliminated. LinRegPCR output from two plates of the same panel was then loaded into Factor-qPCR^37^ (version 2020.0) to remove systematic bias between different RT-qPCR runs. Finally, arithmetic mean of technical replicates was taken as the Cq value representing biological samples.

### Expression stability analysis

Gene expression stability was assessed using RefFinder^40^ (https://www.heartcure.com.au/reffinder/), a web-based tool integrating four algorithms: BestKeeper^25^, DeltaCq^26^, geNorm^18^ and NormFinder^27^. The standalone software of geNorm (qBase + ^34^ from Biogazelle) and NormFinder (R-based, version 5) were used in parallel. For all software, input data was Cq values, output were different types of stability values (BestKeeper: r, DeltaCq: Mean SD of mean $\Delta$Cq, geNorm: M, NormFinder: ρ) and stability rankings. A lower rank indicates a more stable gene. An overall ranking was determined for each gene by calculating geometric mean of rankings from all 4 methods.

### Relative expression of *Ttr *and *Otx2*, statistical analysis and data visualisation

For expression analysis of *Ttr*, geometric mean of P0 samples was used as Calibrator. For expression analysis of *Otx2,* geometric mean of Ctrl samples was used as Calibrator. Relative expression level was calculated by Pfaffl method^64^ (or efficiency method), which uses amplification efficiency E and difference of cycle of quantification $\Delta$Cq from unknown samples and Calibrator.

To evaluate statistical difference in target genes’ RNA level between different groups, one-way ANOVA followed by Tukey’s multiple comparisons was applied. Statistical analysis and graphs construction were performed in GraphPad Prism (version 8.4.0). Data was presented as Mean ± SD.

## Supplementary Information

Supplementary Information.

## References

[1] Dani, N. *et al.* A cellular and spatial map of the choroid plexus across brain ventricles and ages. *bioRxiv* (2019) 10.1101/627539. 10.1016/j.cell.2021.04.003 PMC8214809 33932339

[2] Sturrock RR (1979). A morphological study of the development of the mouse choroid plexus. J. Anat..

[3] Wilting J, Christ B (1989). An experimental and ultrastructural study on the development of the avian choroid plexus. Cell Tissue Res..

[4] Redzic ZB, Preston JE, Duncan JA, Chodobski A, Szmydynger-Chodobska J (2005). The choroid plexus-cerebrospinal fluid system: from development to aging. Curr. Top. Dev. Biol..

[5] Damkier HH, Brown PD, Praetorius J (2013). Cerebrospinal fluid secretion by the choroid plexus. Physiol. Rev..

[6] Strazielle N, Ghersi-Egea JF (2013). Physiology of blood-brain interfaces in relation to brain disposition of small compounds and macromolecules. Mol. Pharm..

[7] Myung J (2018). The choroid plexus is an important circadian clock component. Nat. Commun..

[8] Quintela T (2018). The choroid plexus harbors a circadian oscillator modulated by estrogens. Chronobiol. Int..

[9] Spatazza J (2013). Choroid-plexus-derived Otx2 homeoprotein constrains adult cortical plasticity. Cell Rep..

[10] Balusu S, Brkic M, Libert C, Vandenbroucke RE (2016). The choroid plexus-cerebrospinal fluid interface in Alzheimer’s disease: more than just a barrier. Neural Regener. Res..

[11] Cui J (2020). Inflammation of the embryonic choroid plexus barrier following maternal immune activation. Dev. Cell.

[12] Shen MD (2017). Increased extra-axial cerebrospinal fluid in high-risk infants who later develop autism. Biol. Psychiatry.

[13] Palha JA (2012). Do genes and environment meet to regulate cerebrospinal fluid dynamics? Relevance for schizophrenia. Front. Cell. Neurosci..

[14] Martirosian, V., Julian, A. & Neman, J. The Role of the Choroid Plexus in the Pathogenesis of Multiple Sclerosis. in *The Choroid Plexus and Cerebrospinal Fluid: Emerging Roles in CNS Development, Maintenance, and Disease Progression* 103–127 (Elsevier Inc., 2016). 10.1016/B978-0-12-801740-1.00007-X.

[15] Lowe R, Shirley N, Bleackley M, Dolan S, Shafee T (2017). Transcriptomics technologies. PLoS Comput. Biol..

[16] Bustin SA (2009). The MIQE guidelines: minimum information for publication of quantitative real-time PCR experiments. Clin. Chem..

[17] VanGuilder HD, Vrana KE, Freeman WM (2008). Twenty-five years of quantitative PCR for gene expression analysis. Biotechniques.

[18] Vandesompele J (2002). Accurate normalization of real-time quantitative RT-PCR data by geometric averaging of multiple internal control genes. Genome Biol..

[19] Warrington JA, Nair A, Mahadevappa M, Tsyganskaya M (2000). Comparison of human adult and fetal expression and identification of 535 housekeeping/maintenance genes. Physiol. Genomics.

[20] Gomes AÉI (2018). Selection and validation of reference genes for gene expression studies in Klebsiella pneumoniae using Reverse Transcription Quantitative real-time PCR. Sci. Rep..

[21] Coulson DTR (2008). Identification of valid reference genes for the normalization of RT qPCR gene expression data in human brain tissue. BMC Mol. Biol..

[22] Cheung TT, Weston MK, Wilson MJ (2017). Selection and evaluation of reference genes for analysis of mouse (Mus musculus) sex-dimorphic brain development. PeerJ.

[23] Ramhøj L, Axelstad M, Svingen T (2019). Validation of endogenous reference genes in rat cerebral cortex for RT-qPCR analyses in developmental toxicity studies. PeerJ.

[24] Panina Y, Germond A, Masui S, Watanabe TM (2018). Validation of common housekeeping genes as reference for qPCR gene expression analysis during iPS reprogramming process. Sci. Rep..

[25] Pfaffl MW, Tichopad A, Prgomet C, Neuvians TP (2003). Determination of stable housekeeping genes, differentially regulated target genes and sample integrity: BestKeeper - Excel-based tool using pair-wise correlations. Biotechnol. Lett..

[26] Silver N, Best S, Jiang J, Thein SL (2006). Selection of housekeeping genes for gene expression studies in human reticulocytes using real-time PCR. BMC Mol. Biol..

[27] Andersen CL, Jensen JL, Ørntoft TF (2004). Normalization of real-time quantitative reverse transcription-PCR data: a model-based variance estimation approach to identify genes suited for normalization, applied to bladder and colon cancer data sets. Cancer Res..

[28] Herbert J (1986). Transthyretin: A choroid plexus-specific transport protein in human brain. The 1986 S. Weir Mitchell Award. Neurology.

[29] Planques, A., Oliveira Moreira, V., Dubreuil, C., Prochiantz, A. & Di Nardo, A. A. OTX2 Signals from the Choroid Plexus to Regulate Adult Neurogenesis. *eNeuro*. 10.1523/ENEURO.0262-18.2019 (2019). 10.1523/ENEURO.0262-18.2019 PMC6506823 31064838

[30] Rydbirk R (2016). Assessment of brain reference genes for RT-qPCR studies in neurodegenerative diseases. Sci. Rep..

[31] Dheda K (2004). Validation of housekeeping genes for normalizing RNA expression in real-time PCR. Biotechniques.

[32] Ye J (2012). Primer-BLAST: A tool to design target-specific primers for polymerase chain reaction. BMC Bioinformatics.

[33] Dutta S, Sengupta P (2016). Men and mice: relating their ages. Life Sci..

[34] Hellemans J, Mortier G, De Paepe A, Speleman F, Vandesompele J (2007). qBase relative quantification framework and software for management and automated analysis of real-time quantitative PCR data. Genome Biol..

[35] Lefever S (2009). RDML: structured language and reporting guidelines for real-time quantitative PCR data. Nucleic Acids Res..

[36] Ruijter JM (2009). Amplification efficiency: linking baseline and bias in the analysis of quantitative PCR data. Nucleic Acids Res..

[37] Ruijter, J. M., Ruiz Villalba, A., Hellemans, J., Untergasser, A. & van den Hoff, M. J. B. Removal of between-run variation in a multi-plate qPCR experiment. *Biomol. Detect. Quantif.* **5**, 10–14 (2015). 10.1016/j.bdq.2015.07.001 PMC4822202 27077038

[38] Johnson G, Nolan T, Bustin SA (2013). Real-time quantitative PCR, pathogen detection and MIQE. Methods Mol. Biol..

[39] Allen Institute for Brain Science. *Allen Mouse Brain Atlas* https://mouse.brain-map.org (2004).

[40] Xie F, Xiao P, Chen D, Xu L, Zhang B (2012). miRDeepFinder: A miRNA analysis tool for deep sequencing of plant small RNAs. Plant Mol. Biol..

[41] Krishnan V, Id S, Sampathkumar NK, Massaad C (2019). Optimal use of statistical methods to validate reference gene stability in longitudinal studies. PLoS ONE.

[42] Kirschneck C (2017). Valid gene expression normalization by RT-qPCR in studies on hPDL fibroblasts with focus on orthodontic tooth movement and periodontitis. Sci. Rep..

[43] Hruz T (2011). RefGenes: identification of reliable and condition specific reference genes for RT-qPCR data normalization. BMC Genomics.

[44] Sugiyama S (2008). Experience-dependent transfer of Otx2 homeoprotein into the visual cortex activates postnatal plasticity. Cell.

[45] Spatazza J (2013). Choroid-plexus-derived Otx2 homeoprotein constrains adult cortical plasticity. Cell Rep..

[46] Apulei J (2017). Non-cell autonomous OTX2 homeoprotein regulates visual cortex plasticity through Gadd45. Celeb Cortex.

[47] Beurdeley M (2012). Otx2 binding to perineuronal nets persistently regulates plasticity in the mature visual cortex. J. Neurosci..

[48] Lun MP (2015). Spatially Heterogeneous Choroid Plexus Transcriptomes Encode Positional Identity and Contribute to Regional CSF Production. Neuroscience.

[49] Ge R (2017). Choroid plexus-cerebrospinal fluid route for monocyte-derived macrophages after stroke. J. Neuroinflammation.

[50] Prasongchean W, Vernay B, Asgarian Z, Jannatul N, Ferretti P (2015). The neural milieu of the developing choroid plexus: neural stem cells, neurons and innervation. Front. Neurosci..

[51] Akhtar W, Veenstra GJC (2011). TBP-related factors: a paradigm of diversity in transcription initiation. Cell Biosci..

[52] Uechi T, Tanaka T, Kenmochi N (2001). A complete map of the human ribosomal protein genes: Assignment of 80 genes to the cytogenetic map and implications for human disorders. Genomics.

[53] Yuan M (2014). Selection and evaluation of potential reference genes for gene expression analysis in the brown planthopper, Nilaparvata lugens (Hemiptera: Delphacidae) using reverse-transcription quantitative PCR. PLoS ONE.

[54] Carbajo RJ (2005). Structure of the F1-binding domain of the stator of bovine F1Fo-ATPase and how it binds an α-subunit. J. Mol. Biol..

[55] Zhao T, Mu X, You Q (2017). Succinate: An initiator in tumorigenesis and progression. Oncotarget.

[56] Bowler MW (2013). Conformational dynamics in phosphoglycerate kinase, an open and shut case?. FEBS Lett..

[57] Zhang JY (2015). Critical protein GAPDH and its regulatory mechanisms in cancer cells. Cancer Biol. Med..

[58] Plano SA (2017). Circadian and metabolic effects of light: Implications in weight homeostasis and health. Frontiers Neurol..

[59] Sieber MH, Spradling AC (2017). The role of metabolic states in development and disease. Curr. Opin. Genet. Dev..

[60] Bittermann E (2019). Differential requirements of tubulin genes in mammalian forebrain development. PLoS Genet..

[61] Gasic I, Boswell SA, Mitchison TJ (2019). Tubulin mRNA stability is sensitive to change in microtubule dynamics caused by multiple physiological and toxic cues. PLoS Biol..

[62] Panina Y, Germond A, Masui S, Watanabe TM (2018). Validation of Common Housekeeping Genes as Reference for qPCR Gene Expression Analysis during iPS Reprogramming Process. Sci. Rep..

[63] Strazielle N, Ghersi-Egea JF (1999). Demonstration of a coupled metabolism-efflux process at the choroid plexus as a mechanism of brain protection toward xenobiotics. J. Neurosci..

[64] Pfaffl MW (2001). A new mathematical model for relative quantification in real-time RT-PCR. Nucleic Acids Res..

[65] Yan Z (2016). Quantitative evaluation and selection of reference genes for quantitative RT-PCR in mouse acute pancreatitis. Biomed Res. Int.

[66] Nagdas S (2019). Drp1 promotes KRas-driven metabolic changes to drive pancreatic tumor growth. Cell Rep..

[67] Khimani AH (2005). Housekeeping genes in cancer: normalization of array data. Biotechniques.

[68] Eissa N, Kermarrec L, Hussein H, Bernstein CN, Ghia JE (2017). Appropriateness of reference genes for normalizing messenger RNA in mouse 2,4-dinitrobenzene sulfonic acid (DNBS)-induced colitis using quantitative real time PCR. Sci. Rep..

[69] Massimino L (2018). TBR2 antagonizes retinoic acid dependent neuronal differentiation by repressing Zfp423 during corticogenesis. Dev. Biol..

[70] Hooshmand MJ (2017). Neutrophils induce astroglial differentiation and migration of human neural stem cells via C1q and C3a synthesis. J. Immunol..

[71] Moy JK, Khoutorsky A, Asiedu MN, Dussor G, Price TJ (2018). eIF4E phosphorylation influences Bdnf mRNA translation in mouse dorsal root ganglion neurons. Front. Cell. Neurosci..

[72] Kutscher, L. M. *et al.* Functional loss of a noncanonical BCOR-PRC1.1 complex accelerates SHH-driven medulloblastoma formation. *Genes Dev.* **34**, 1161–1176 (2020). 10.1101/gad.337584.120 PMC7462063 32820036

[73] Campla CK (2019). Targeted deletion of an NRL- and CRX-regulated alternative promoter specifically silences FERM and PDZ domain containing 1 (Frmpd1) in rod photoreceptors. Hum. Mol. Genet..

[74] Friedman JS (2006). Premature truncation of a novel protein, RD3, exhibiting subnuclear localization is associated with retinal degeneration. Am. J. Hum. Genet..

[75] Spandidos A, Wang X, Wang H, Seed B (2009). PrimerBank: a resource of human and mouse PCR primer pairs for gene expression detection and quantification. Nucleic Acids Res..

[76] Thomas KC (2014). Evidence based selection of commonly used RT-qPCR reference genes for the analysis of mouse skeletal muscle. PLoS ONE.

[77] Carlin, D., Halevi, A. E., Ewan, E. E., Moore, A. M. & Cavalli, V. Nociceptor Deletion of Tsc2 Enhances Axon Regeneration by Inducing a Conditioning Injury Response in Dorsal Root Ganglia. *eNeuro* (2019). Doi: 10.1523/ENEURO.0168-19.2019 10.1523/ENEURO.0168-19.2019 PMC6595439 31182472

[78] Oh YM (2018). Epigenetic regulator UHRF1 inactivates REST and growth suppressor gene expression via DNA methylation to promote axon regeneration. Proc. Natl. Acad. Sci. U. S. A..

[79] Musilli S (2017). DNA damage induced by Strontium-90 exposure at low concentrations in mesenchymal stromal cells: the functional consequences. Sci. Rep..

[80] Perry S (2019). Characterization of Dmrt3-derived neurons suggest a role within locomotor circuits. J. Neurosci..

[81] Chutake YK (2015). FXN promoter silencing in the humanized mouse model of friedreich ataxia. PLoS ONE.

[82] Pallotta MM (2017). Specific effects of chronic dietary exposure to chlorpyrifos on brain gene expression: a mouse study. Int. J. Mol. Sci..

[83] Scicchitano S (2019). The stem cell-associated transcription co-factor, ZNF521, interacts with GLI1 and GLI2 and enhances the activity of the Sonic hedgehog pathway. Cell Death Dis..

[84] Yoshikawa A, Nakamachi T, Shibato J, Rakwal R, Shioda S (2014). Comprehensive analysis of neonatal versus adult unilateral decortication in a mouse model using behavioral, neuroanatomical, and DNA microarray approaches. Int. J. Mol. Sci..

[85] Cosway EJ (2017). Redefining thymus medulla specialization for central tolerance. J. Exp. Med..

[86] Jiang Y (2014). A neurostimulant para-chloroamphetamine inhibits the arginylation branch of the N-end rule pathway. Sci. Rep..

[87] Yang, H. *et al.* Generation of functional dopaminergic neurons from human spermatogonial stem cells to rescue parkinsonian phenotypes. Stem Cell Res. Therapy. 10.1186/s13287-019-1294-x. 10.1186/s13287-019-1294-x PMC6598262 31248447

[88] Cho J (2015). Multiple repressive mechanisms in the hippocampus during memory formation. Science.

