# Supplementary material for: Assessment of common housekeeping genes as reference for gene expression studies using RT-qPCR in mouse choroid plexus
Source: Sci Rep. 2021 Feb 8;11:3278. doi: 10.1038/s41598-021-82800-5 (PMC7870894; doi:10.1038/s41598-021-82800-5)
Supplement: Supplementary file 1 — Supplementary Information. [file 41598_2021_82800_MOESM1_ESM.docx]

Supplementary Information for the publication:

**Assessment of common housekeeping genes as reference for gene expression studies using RT-qPCR in mouse choroid plexus**

Kim Hoa Ho^1,2^, Annarita Patrizi^1,*^

^1^Schaller Research Group, German Cancer Research Center (DKFZ), Heidelberg, Germany

^2^Faculty of Biosciences, Heidelberg University, Heidelberg, Germany

*Corresponding author:

Annarita Patrizi, PhD

[a.patrizi@dkfz-heidelberg.de](mailto:a.patrizi@dkfz-heidelberg.de)

**Table S1.** Overview of experimental conditions, biological and technical replicates

| **Condition** | **Pooled samples per biological replicate** | **Biological replicate** | **Technical replicate** |
| --- | --- | --- | --- |
| ***Developmental panel*** | | | |
| P0 | 2 | 5 | 3 |
| P15 | 2 | 5 | 3 |
| P30 | 1 | 5 | 3 |
| P60 | 1 | 5 | 3 |
| ***Light/Dark rearing panel*** | | | |
| Ctrl | 1 | 5 | 3 |
| D | 1 | 5 | 3 |
| D-1hL | 1 | 5 | 3 |
| D-4hL | 1 | 5 | 3 |
| D-24hL | 1 | 5 | 3 |

**Table S2.** Comparison of geNorm and NormFinder results calculated with original software and RefFinder.

| **Developmental panel** | | | | | | | | |
| --- | --- | --- | --- | --- | --- | --- | --- | --- |
| **Ranking** | **geNorm** | | | | **NormFinder** | | | |
|  | **Original software** | | **RefFinder** | | **Original software** | | **RefFinder** | |
|  | **Gene** | **Stability value** | **Gene** | **Stability value** | **Gene** | **Stability value** | **Gene** | **Stability value** |
| 1 | *Rpl27* | 0.098 | *Rpl27* | 0.07 | *Gapdh* | 0.161 | *Gapdh* | 0.125 |
| 2 | *Rpl13a* | 0.104 | *Rpl13a* | 0.07 | *Rer1* | 0.207 | *Rer1* | 0.162 |
| 3 | *Actb* | 0.115 | *Actb* | 0.115 | *Ubc* | 0.248 | *Ubc* | 0.231 |
| 4 | *Tbp* | 0.156 | *Tbp* | 0.156 | *Atp5f1* | 0.261 | *Atp5f1* | 0.245 |
| 5 | *Rer1* | 0.216 | *Rer1* | 0.216 | *B2m* | 0.278 | *B2m* | 0.272 |
| 6 | *Ubc* | 0.258 | *Ubc* | 0.258 | *Rpl13a* | 0.309 | *Rpl13a* | 0.361 |
| 7 | *Gapdh* | 0.312 | *Gapdh* | 0.312 | *Rpl27* | 0.314 | *Rpl27* | 0.366 |
| 8 | *Atp5f1* | 0.349 | *Atp5f1* | 0.349 | *Actb* | 0.351 | *Actb* | 0.386 |
| 9 | *B2m* | 0.376 | *B2m* | 0.376 | *Pgk1* | 0.363 | *Pgk1* | 0.399 |
| 10 | *Pgk1* | 0.41 | *Pgk1* | 0.41 | *Tbp* | 0.438 | *Tbp* | 0.492 |
| 11 | *Sdha* | 0.452 | *Sdha* | 0.452 | *Sdha* | 0.469 | *Sdha* | 0.54 |
| 12 | *Hprt1* | 0.48 | *Hprt1* | 0.48 | *Hprt1* | 0.473 | *Hprt1* | 0.568 |
| **Light/Dark rearing panel** | | | | | | | | |
| **Ranking** | **geNorm** | | | | **NormFinder** | | | |
|  | **Original software** | | **RefFinder** | | **Original software** | | **RefFinder** | |
|  | **Gene** | **Stability value** | **Gene** | **Stability value** | **Gene** | **Stability value** | **Gene** | **Stability value** |
| 1 | *Hprt1* | 0.115 | *Hprt1* | 0.113 | *Hprt1* | 0.121 | *Hprt1* | 0.139 |
| 2 | *Rpl13a* | 0.115 | *Rpl13a* | 0.113 | *Atp5f1* | 0.123 | *Tbp* | 0.149 |
| 3 | *Rpl27* | 0.121 | *Rpl27* | 0.127 | *Tbp* | 0.132 | *Gapdh* | 0.154 |
| 4 | *Rer1* | 0.135 | *Rer1* | 0.14 | *Rpl27* | 0.135 | *Rpl27* | 0.154 |
| 5 | *Gapdh* | 0.182 | *Tbp* | 0.184 | *Gapdh* | 0.151 | *Atp5f1* | 0.179 |
| 6 | *Tbp* | 0.205 | *Gapdh* | 0.206 | *Rpl13a* | 0.157 | *Rpl13a* | 0.185 |
| 7 | *Atp5f1* | 0.22 | *Atp5f1* | 0.22 | *Pgk1* | 0.163 | *Pgk1* | 0.198 |
| 8 | *Ubc* | 0.231 | *Ubc* | 0.232 | *Ubc* | 0.164 | *Ubc* | 0.211 |
| 9 | *Pgk1* | 0.24 | *Pgk1* | 0.24 | *Actb* | 0.166 | *Rer1* | 0.224 |
| 10 | *Actb* | 0.253 | *Actb* | 0.253 | *Rer1* | 0.189 | *Actb* | 0.231 |
| 11 | *Sdha* | 0.264 | *Sdha* | 0.264 | *Sdha* | 0.19 | *Sdha* | 0.25 |
| 12 | *B2m* | 0.279 | *B2m* | 0.278 | *B2m* | 0.212 | *B2m* | 0.294 |


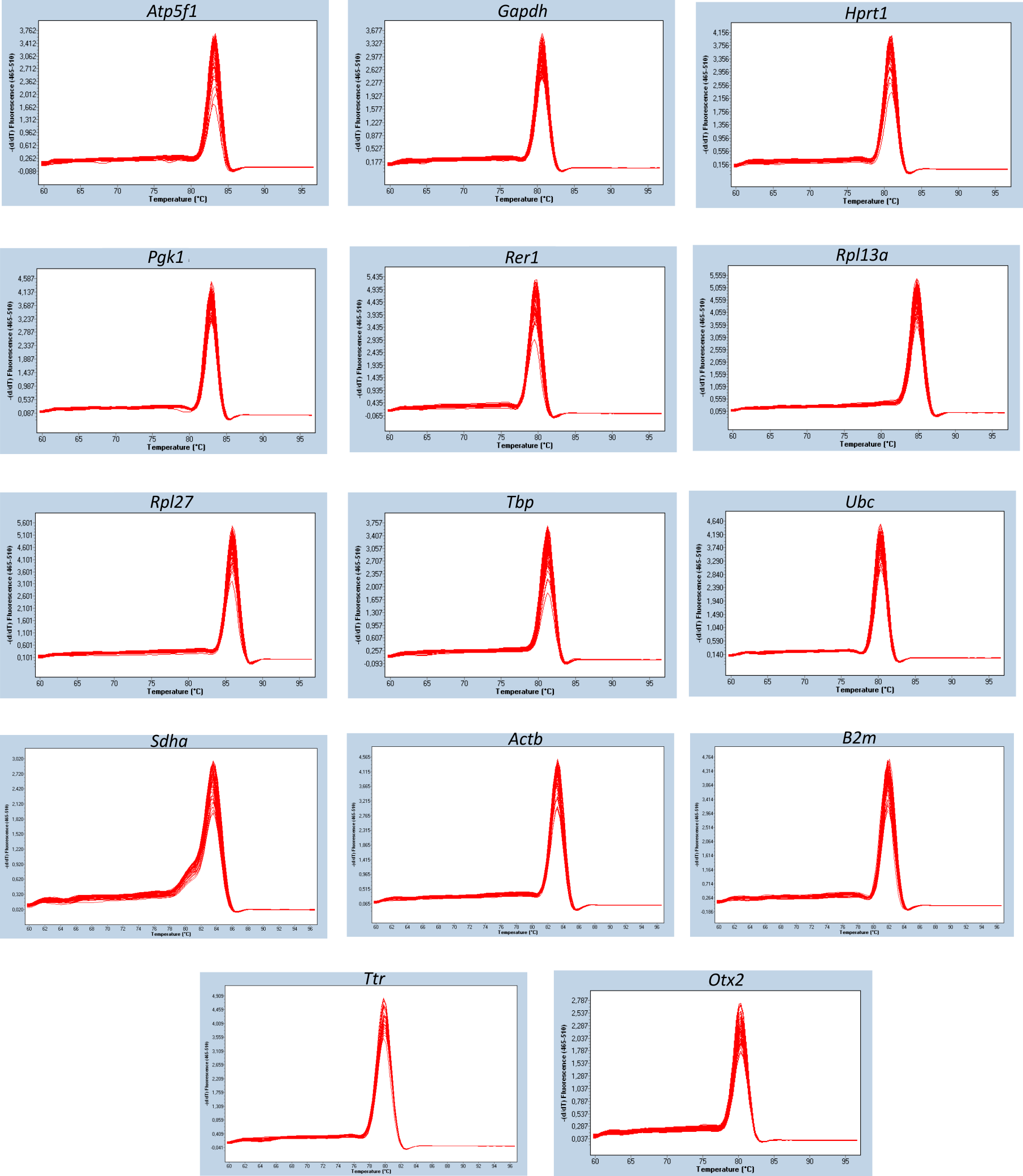
**Figure S1.**

**qPCR melting curve analysis**. The primers specificity for 12 candidate reference genes and 2 target genes was determined by melting-curve analysis. Data generated by Roche LightCycler® 480.

**Figure S2.**


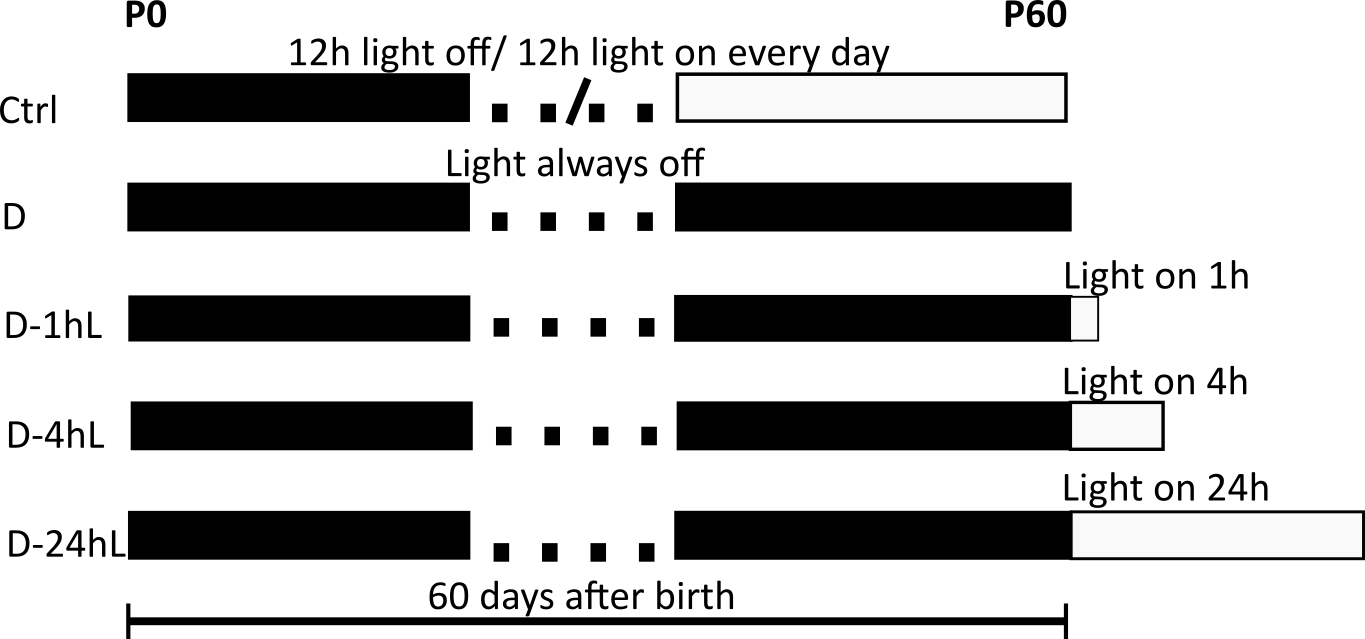


**Experimental paradigm for the Light/Dark rearing panel**. Control mice (Ctrl) were reared in normal condition of 12 hours light/dark cycles. Dark reared mice (D) were reared in the dark from birth until P60. D-1hL mice were re-exposed to light for 1 hour before being sacrificed; D-4hL and D-24hL were D mice exposed to light for 4 hour and 24 hour respectively.

**Figure S3.**

**
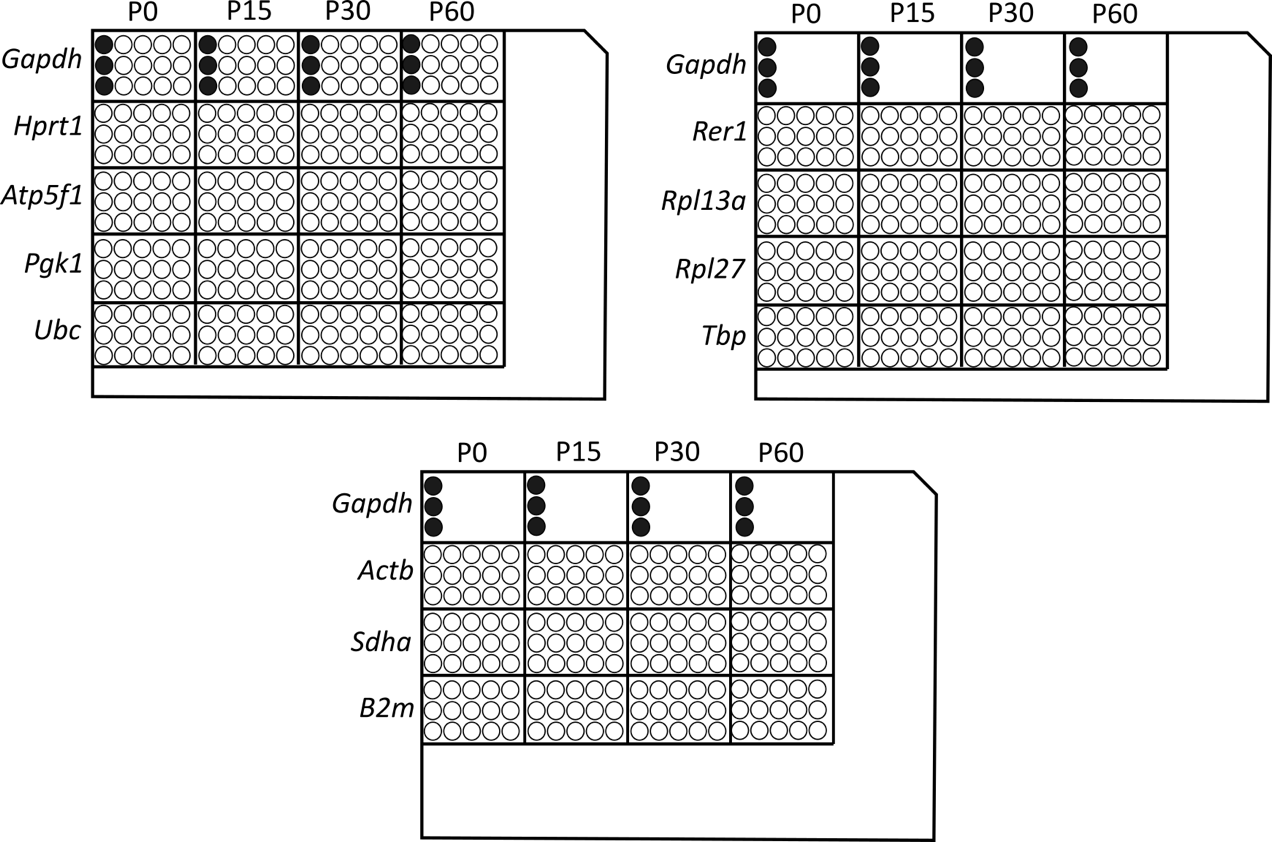
**

**Experimental setup following sample maximization approach**. An example of simplified layout for Developmental panel. One sample from each age group was used with *Gapdh* primers (black dots) to provide inter-run calibrators (IRC) for downstream correction.

**Figure S4.**


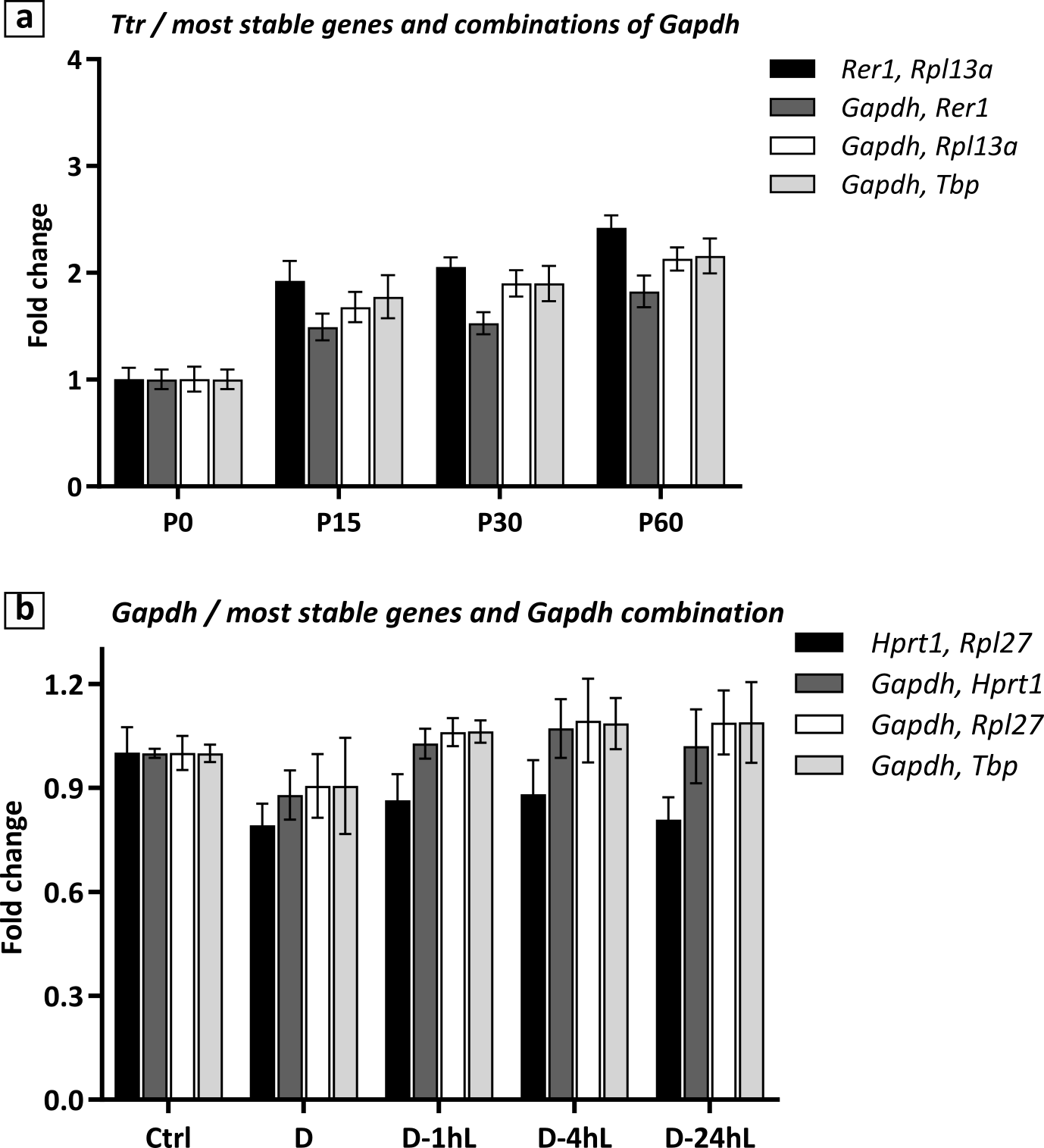


**The effect of combining 1 more stable gene to *Gapdh* as normalisation factor** (**a**) *Ttr* relative expression in the Developmental panel normalized to *Rer1, Rpl13a* versus different combination of *Gapdh* with more stable genes, as *Rer1, Rpl13a, Tbp.* (**b**) *Otx2* relative expression in the Light/Dark rearing panel normalized to *Hprt1, Rpl27* versus different combination of *Gapdh* with more stable genes, as *Hprt1, Rpl27, Tbp.* Data is presented as Mean ± SD.
